# Is Cross-Shareholding Conducive to Corporate Sustainability? Evidence From the Environmental Investment of Chinese Listed Firms

**DOI:** 10.3389/fpsyg.2021.789811

**Published:** 2021-11-05

**Authors:** Jinfang Tian, Wei Cao, Xuzhao Ji

**Affiliations:** School of Statistics, Shandong University of Finance and Economics, Jinan, China

**Keywords:** cross-shareholding, environmental investment, corporate sustainable development, emerging market, China

## Abstract

This article examines the impact of cross-shareholding on corporate environmental investment (Env) using Chinese listed firms from 2014 to 2019 as the research setting. The results show that there is a positive impact of cross-shareholding on corporate environmental investment. The finding remains robust to a battery of robustness checks. In addition, the heterogeneity analysis illustrates that the positive impact of cross-shareholding on corporate environmental investment is more pronounced in state-owned firms and high-polluting industries when compared to non-state-owned firms and low-polluting industries, respectively. This study extends the research on cross-shareholding and provides practical implications for corporate sustainable development.

## Introduction

Inter-firm cross-shareholding is when two or more firms hold shares in each other’s firms, entailing a binding of financial interests. Its main purposes are to reduce transaction risks (Williamson, 1979), resist hostile takeovers (Nyberg, 1995), and increase profits (Amundsen and Beergman, 2002). Cross-shareholding between firms can bring a range of synergistic benefits such as improving information advantages, facilitating inter-firm collaborations, and fulfilling financing demands (Uzzi, 1999; Rauch and Casella, 2003; Cohen et al., 2008).

Due to the increasing complexity and volatility of stock market, cross-shareholding has become a popular mode for listed companies in China to maintain their market competitiveness (Peng et al., 2019; Guo H. et al., 2021). The popularity of inter-firm cross-shareholding in China’s capital market is further fueled by the country’s vigorous shareholding reform, continuous stock market expansion, rising demand for capital operation, and the arbitrage motives of short-term capital flows (Peng et al., 2019).

Corporate environmental investment (Env) refers to companies’ practices and initiatives to help protect the environment (Nakamura, 2011). Companies are the primary resource consumer and polluter (Tian et al., 2020), and as such, they are obliged to assume responsibilities for environmental governance (Wan et al., 2021). Environmental regulations, as a major part of China’s green development efforts, have also compelled businesses to reduce the damage to the environment during their production process. Firms have an essential role in environmental protection, and corporate environmental investment is crucial to facilitating the green development of society (Li et al., 2021). Existing studies are concentrated around the impacts of policies and within-firm factors on corporate environmental investment (Saltari and Travaglini, 2011; Wei and Zhou, 2020; Huang and Lei, 2021), the impacts of inter-firm factors are seldom investigated. Therefore, this article aims to examine the impact of cross-shareholding on corporate environmental investment.

Corporate strategy is one of the main internal factors affecting firms’ environmental investment decisions (Wei and Zhou, 2020). Cross-shareholding strategy has the potential to make contributions to corporate healthy development (Liu et al., 2018); its relationship with corporate environmental investment is thus worth studying. This article selects Chinese listed firms as the research setting for the following reasons. First, China’s carbon emissions per unit of GDP surpass the global average, and its carbon emissions in 2019 reached nearly 10 billion tons, ranking first in the world (The World Bank, 2016; BP, 2019). Second, since businesses are the main resource consumer and polluter, they are obliged to take environmental responsibility (Fan et al., 2021). Third, the strong emphasis on social relationships and networking in Chinese culture (Yan and Sun, 2021), as well as the weak regulation of cross-shareholding in Chinese corporate law, both contribute to the rising popularity of cross-shareholding among Chinese firms. However, there has been little research on the impact of cross-shareholding on corporate environmental investment.

Therefore, this article examines the impact of cross-shareholding on corporate environmental investment using Chinese listed companies as the research setting. The results indicate that cross-shareholding has a positive impact on corporate environmental investment. Further analysis shows a heterogeneous effect of corporate ownership structure and industry effect in the relationship between cross-shareholding and corporate environmental investment. The positive effect of cross-shareholding on corporate environmental investment is more pronounced in state-owned companies or firms in the heavily polluting industry. The results remain robust when using alternative measure of the cross-shareholding variable and using random sampling approach.

The remainder of the article is structured as follows. In section “Literature Review and Hypothesis Development,” we review the prior research on cross-shareholding and environmental investment, and propose the hypotheses. Section “Research Design” describes the data and variables. In section “Results,” regression analysis is conducted to examine the hypotheses, followed by heterogeneity analysis and robustness checks. Section “Conclusion” concludes the article.

## Literature Review and Hypothesis Development

### Cross-Shareholding

Inter-firm cross-shareholding is the practice of two or more firms holding shares in each other’s firms. Companies create business alliances through cross-shareholding, which helps them share resources, reduce production costs and expand production scale (Ranjan, 1998; Boyatzis et al., 2015), and improve financial performance and corporate governance (Farrell and Shapiro, 1988; Ranjan, 1998). Cross-shareholding is classified into two types: one-way cross-shareholding and two-way cross-shareholding. The cross-shareholding models in China are predominantly one-way cross-shareholding (Guo H. et al., 2021); therefore, this article defines the concept of cross-shareholding using one-way cross-shareholding, i.e., firm A holds shares of firm B, but firm B is not required to hold shares of firm A at the same time (Flath, 1992).

Cross-shareholding is mainly used to reduce operation risks (Williamson, 1979), resist hostile takeovers (Nyberg, 1995), and increase financial returns (Amundsen and Beergman, 2002). The special inter-firm relationship of cross-shareholding can help overcome certain flaws of external mechanisms, which is critical to China’s economic transformation (Peng et al., 2019; Bourgeois-Bougrine, 2020). Inter-firm cross-shareholding has become very popular in Chinese capital market due to China’s active shareholding reform, continuous stock market expansion, rising demand for capital operations, and the arbitrage motivations of short-term capital flow (Peng et al., 2019). Another possible explanation for the rise of cross-shareholding in China is that the society places a high value on social relationships and networking (Xue et al., 2021), and so corporate finance happens to be highly dependent on social ties (Talavera et al., 2012; Su et al., 2020). Inter-firm cross-shareholding has a relatively strong synergistic effect among Chinese firms in terms of enhancing information advantages (Cohen et al., 2008), corporate cooperation (Uzzi, 1999), and financing capacity (Rauch and Casella, 2003).

Through cross-shareholding, listed firms can form a stable strategic alliance with equity ties (Gibb and Li, 2003), which allows them to share resources, reduce production costs and expand production scale, achieve economies of scale (Ranjan, 1998; Park and Luo, 2010) and improve corporate governance (Farrell and Shapiro, 1988; Ranjan, 1998), ultimately improving financial performance (Singh and Delios, 2017). At the same time, cross-shareholding can protect firms from hostile takeovers, reduce risks, and increase profits. Firms that cross-hold shares can not only earn dividends from equity, but also achieve higher financial performance as a result of industry chain integration and complementary advantages (Brooks et al., 2018).

### Corporate Environmental Investment

Environmental investment refers to the total expenses related to environmental practices such as pollution control and environment improvement, which belong to a special type of corporate investment (Ehresman and Okereke, 2015). Environmental investment pursues economic, environmental, and social returns, but the latter two tend to outweigh the economic returns (Ehresman and Okereke, 2015). Environmental investments do not generate direct capital inflows, and they often require significant extra expenditure on environmental facilities and technologies, leaving firms with little incentives to practice (Orsato, 2006). Based on factor endowment hypothesis, corporate environmental investment decisions are the tradeoff between costs and returns (Leiter et al., 2011). Firms thus tend to lack motivations to make voluntary environmental investments. However, firms can benefit from investing in pro-environmental activities. On the one hand, higher environmental investment implies a reduction in the cost of environmental compliance (Maxwell and Decker, 2006). On the other hand, firms enjoy better reputation by delivering a positive and environmentally friendly image to the public (Wei and Zhou, 2020).

Corporate environmental investment is susceptible to both external and internal factors. The external factors primarily include the degree of government intervention and institutional constraint, and regional economic development (Saltari and Travaglini, 2011; Ducassy and Montandrau, 2015; Huang and Lei, 2021). Internal factors are mainly corporate financial performance (Blanco et al., 2009) and internal governance (Wei and Zhou, 2020); for example, a healthy financial position makes it easy for businesses to make environmental investments (Blanco et al., 2009).

### Hypothesis Development

Cross-shareholding is critical for improving corporate performance. Through cross-shareholding, a strategic alliance with equity ties can be formed between firms, which helps firms to reduce production costs and expand production scale through information sharing and technology complementation, thus achieving economies of scale (Ranjan, 1998) and higher financial performance (Nyberg, 1995). Moreover, cross-shareholding shields firms from hostile takeovers while simultaneously lower risks and increase profits. Firms can benefit not just from dividends generated from cross-holding stocks, but also from improved financial performance as a result of industry chain integration or complementary advantages (Brooks et al., 2018). Corporate environmental investment is commercial investment; thus, firms’ financial performance will have a direct impact on the scale of their environmental investment, and a healthy financial position is helpful in promoting environmental investment (Blanco et al., 2009).

Furthermore, cross-shareholding is conducive to reducing managerial myopia and speculative behaviors (Gilson and Roe, 1993; Guo L. X. et al., 2021) and so encouraging firms to pay more attention to long-term benefits. Managers are more inclined to make environmental investments when the purpose is to maintain corporate reputation, social image, and sustainable development. And the reduction of management speculative behavior can lead to more compliant business operations (Rocha and Salomão, 2019). When firms are under stringent government environmental regulations, they are incentivized to reduce environmental compliance costs by increasing environmental investment (Maxwell and Decker, 2006; Bierbaum et al., 2020).

Taken together, cross-shareholding may increase corporate environmental investment by enhancing financial performance and reducing managerial myopia and speculative behaviors. Based on this, this article proposes the following hypothesis:

*Hypothesis 1: Cross-shareholding has a positive impact on corporate environmental investment.*

Although firms play an important role in social and environmental development, their incentives to fulfill environmental responsibility might change as their ownership structure shifts. In China, state-owned enterprises (SOEs) are closely tied to the government and they control the bulk of economic resources (Li and Wang, 2021). However, besides economic responsibility, SOEs are expected to take on social responsibility as well, and thus they are more susceptible to government policies, particularly strategic and social policies (Lin and Tan, 1999; Xue et al., 2019). Moreover, since government support such as financial and policy support is heavily tilted in favor of SOEs, SOEs suffer considerably less financial pressure than non-SOEs (Lin, 2021). When the state has more protection and supervision over SOEs, they become more susceptible to state intervention (Kornai, 1986). As a result, in the context of China’s active national campaign for low-carbon transition and carbon neutrality, SOEs are more likely to make green investment. Therefore, we propose the following hypothesis:

*Hypothesis 2: Cross-shareholding has a greater influence on environmental investment in SOEs than it does in non-SOEs.*

Firms’ responses to market changes differ by industry, as do government macro control policies (Halme and Huse, 1997). As a major resource consumer and polluter, firms are obliged to take environmental responsibility, which is also reflected in one of China’s environmental policies of ‘‘assigning responsibility to those who created pollution to clearing it up’’^1^. Since heavy polluting industries cause more environmental problems, they are subject to more government oversight, which means higher environmental compliance costs and, as a result, a larger scale of environmental investment (Chang et al., 2021). Chinese government has been aggressively promoting sustainable development by stepping up efforts to preserve the ecological environment and control carbon emissions. The Chinese environmental protection authorities have issued regulations, such as the *Notice on Further Regulating the Environmental Evaluation of Companies Applying for Listing or Refinancing in the Heavy Pollution Industry*^2^ and the *Regulation on Management of Inventory of Pollutant Discharging Units subject to Key Management*^3^, further strengthening the supervision and punishment mechanism for heavy polluting industries. As a result, the heavily polluting industries face much stronger external regulation than low polluting industries (Wang et al., 2021). Therefore, in order to reduce the cost of environmental compliance, heavy polluters are more likely to invest in environmental measures or projects. Based on this, this article proposes the following hypothesis:

*Hypothesis 3: The impact of cross-shareholding on the environmental investment in heavily polluting industries is stronger compared to low polluting industries.*

## Research Design

### Data

This article uses Chinese listed firms in the A-share stock market from 2014 to 2019 as the research setting, of which the cross-shareholding data are sourced from the Wind financial database^4^ and the environmental investment data are retrieved from the accrued expenses related to environmental practices in the notes appended to corporate financial statements. The control variables used in this article are sourced from the China Stock Market & Accounting Research Database (CSMAR)^5^, and the raw data were pre-processed based on the following screening principles: (1) excluding ST, ST^∗^, and delisted firms; (2) excluding samples with missing data from 2014 to 2019. Finally, we reached 1,122 firm-year observations.

### Variables

This section introduces the dependent variable, explanatory variable, and control variables, and presents descriptive statistics and the correlation matrix for all variables as shown in Table 1.

**TABLE 1:** Summary statistics and correlation matrix.

**Variable**	**Mean**	**SD**	**1**	**2**	**3**	**4**	**5**	**6**	**7**	**8**
*1. Env*	16.75	2.24								
*2. Inv*	17.21	2.67	0.25							
*3. Age*	207.12	48.88	−0.07	0.01						
*4. Size*	23.48	1.52	0.54	0.38	−0.07					
*5. Roe*	0.06	0.25	0.07	0.07	0.04	0.09				
*6. Lev*	1.31	7.22	−0.01	−0.03	−0.01	0.06	0.01			
*7. Growth*	0.15	0.71	−0.01	−0.04	0.01	0.03	0.07	−0.02		
*8. First*	0.37	0.16	0.26	0.07	−0.22	0.38	0.00	0.04	0.04	
*9. Cash*	0.13	0.09	−0.14	−0.06	0.02	−0.11	0.10	−0.02	−0.06	−0.08

#### Dependent Variable

Corporate *Env* is the dependent variable. Most of the existing studies on China’s corporate environmental investment use the amount of environmental investment disclosed in the corporate social responsibility (CSR) or sustainability report to represent firms’ environmental investment, but this measurement has certain shortcomings. This is because the Chinese government does not explicitly require listed firms to disclose their environmental investments in their CSR or sustainability reports. When firms choose not to disclose this information, it may result in missing data for the sample firm. Therefore, this article chooses the accrued expenses of wastewater treatment, energy-saving devices, technological upgrading, and related engineering projects as the measurement of corporate environmental investment based on firm’s financial statement notes (Zhang et al., 2019). The financial information of these listed firms are subject to independent third- party audits, which ensures data reliability and precision.

#### Explanatory Variable

Cross-shareholding (*Inv*) is the explanatory variable. This variable denotes the size of firms’ cross-holding investment, which is measured by the natural logarithm of total investment that firms cross-hold in other firms.

#### Control Variables

##### Firm age (*age*)

The longer the firm has been operating, the more likely it is to pay attention to corporate sustainability and invest in environmental projects. This article uses firm age as a control variable, and it is measured by the number of years since the establishment multiplied by 10.

##### Firm size (*size*)

Firms of different sizes has varied abilities to deploy human capital, material, and financial resources, which ultimately affects the scale of environmental investment. This article uses the natural logarithm of total assets to measure the firm size.

##### Profitability (*roe*)

Managers may face varying financial pressure based on their company’s profitability. Although environmental investment enhances corporate sustainability, it can put the company under financial constraints in the short term. Therefore, when corporate profitability is low, managers may reduce environmental investment. In this article, we choose return on assets to measure corporate profitability.

##### Financial leverage (*lev)*

The larger a firm’s financial leverage, the higher the debt risk if faces; and in the face of high debt risk, managers may reduce unnecessary expenses or investments. Therefore, we use total debts divided by total assets to measure the financial leverage.

##### Growth (*growth*)

Corporate growth ability reflects the growth rate of firm size; as firms expand, so does their ability to deploy social resources such as human capital, material, and financial resources; and managers will then deploy commensurate strategies in the continuous expansion, impacting the scale of environmental investment. This article uses the growth rate of operating income to indicate corporate growth ability.

##### Equity concentration (*first*)

Equity concentration can reflect firms’ governance structure effectively which to a certain extent affects corporate strategic decisions. In this article, we use the shares percentage of the largest shareholder to measure equity concentration.

##### Financial constraint (*cash*)

This variable reflects the level of cash flow of sample firms, which directly determines firms’ upper limit for environmental investment. This article uses net cash flow scaled by total assets to measure financial constraint.

## Results

### Baseline Results

This article examines the impact of cross-shareholding on corporate environmental investment using Chinese listed companies from 2014 to 2019 as the research setting, and estimates the following regression. The regression results for the impact of cross-shareholding (*Env*) on corporate environmental investment (*Inv*) are shown in Table 2.


(1) $$\text{Env}=\beta_{0}+\beta_{1}\,I\,n\,v+\beta_{2}\,A\,g\,e+\beta_{3}\,S\,i\,z\,e+\beta_{4}\,R\,o\,e+\beta_{5}\,L\,e\,v+\beta_{6}\,G\,r\,o\,w\,t\,h+\beta_{7}\,F\,i\,r\,s\,t+\beta_{8}\,C\,a\,s\,h+F\,i\,r\,m\,F\,E+Y\,e\,a\,r\,F\,E+\varepsilon$$

**TABLE 2:** Baseline regression results.

**Variable**	**Model (1)**	**Model (2)**	**Model (3)**
*Inv*	0.1034*** (3.22)	0.1053*** (3.27)	0.0879*** (2.77)
*Age*			0.0272*** (4.39)
*Size*			0.7308*** (4.02)
*Roe*			−0.0998 (−0.43)
*Lev*			−0.0131** (−2.28)
*Growth*			−0.072 (−1.12)
*First*			−1.7694* (−1.67)
*Cash*			−1.0001 (−1.14)
*Year FE*	No	Yes	Yes
*Firm FE*	Yes	Yes	Yes
*R-squared*	0.0544	0.0564	0.1181
*N*	1121	1121	1121

(1) *, **, *** represent significant at the 10, 5, and 1% significance level, respectively.

*(2) *t*-values are provided in parentheses.*

Model (1) controls for firm fixed effect (*Firm FE*) with no control variable added. Model (2) adds year fixed effect (*Year FE*) to Model (1). Model (3) adds the control variables of basic firm characteristics, financial indicators and insider control issues, including firm age (*Age)* and firm size (*Size*), profitability (*Roe*), financial leverage (*Lev*), growth ability (*Growth*), financial constraint (*Cash*), and the shares percentage of the largest shareholder (*First*), and controls for firm and year fixed effects.

The results demonstrate that there is a positive and significant impact of cross-shareholding on corporate environmental investment across all regressions. Therefore, cross-shareholding has a positive impact on environmental investment, supporting *Hypothesis 1*.

### Heterogeneity Analysis

#### State Ownership

Environmental investment is a social responsibility and is characterized by long cycle and so low short-term returns, which discourages firms from investing. However, compared to non-SOEs, SOEs are more susceptible to government macro control policies, making them more incentivized to invest in environmental projects (Lin and Tan, 1999). As such, the impact of cross-shareholding on corporate environmental investment may change when firms’ ownership structure changes. To examine the heterogeneity effect of corporate ownership structure in the nexus between cross-shareholding and environmental investment, we introduce the dummy variable *SOE*, which equals to 1 if a firm is state-owned and 0 otherwise. The results are shown in Table 3.

**TABLE 3:** Heterogeneity results for state ownership.

**Variable**	**(1) SOEs**	**(2) Non-SOEs**
*Inv*	0.0979*** (2.64)	0.0685 (1.05)
*Age*	−0.0052*** (−4.51)	−0.0070 (−0.87)
*Size*	0.5227* (2.34)	0.9126** (2.38)
*Roe*	−0.1634 (−0.71)	1.600318 (1.28)
*Lev*	−0.0194*** (−2.91)	0.0021 (0.19)
*Growth*	−0.1267 (−1.22)	−0.0953 (−1.00)
*First*	−1.9540 (−1.60)	−4.1394 (−1.50)
*Cash*	−2.0724* (−1.79)	0.6532 (0.43)
*Year FE*	Yes	Yes
*Firm FE*	Yes	Yes
*R-squared*	0.2814	0.0910
*N*	782	339

(1) *, **, *** represent significant at the 10, 5, and 1% significance level, respectively.

*(2) *t*-values are provided in parentheses.*

The results show that the coefficient of cross-shareholding on corporate environmental investment is significantly positive at the 1% level in SOEs, but not significant in non-SOEs. Therefore, the impact of cross-shareholding on corporate environmental investment is more pronounced in SOEs than in non-SOEs, which supports *Hypothesis 2*.

#### Polluting Industry

To examine the different effects of cross-shareholding on environmental investment in industries with varying level of pollution, we introduce the dummy variable *pollute*, which equals to 1 if a firm falls in the category of heavily polluting industry and 0 otherwise. In terms of the classification criteria, we categorize firms into heavily polluting and low polluting industries based on the *Regulation on Management of Inventory of Pollutant Discharging Units subject to Key Management* (see text footnote 3) issued by the Ministry of Ecology and Environment of the People’s Republic of China, where heavily polluting industries are defined as industries that are subject to priority administration of discharge permits or are generating soluble and highly toxic waste residues, such as thermal power generation, steel manufacturing, non-ferrous metal smelting, mining, textile. Table 4 reports the results.

**TABLE 4:** Heterogeneity results for high versus low polluting industry.

**Variable**	**(1) High polluting**	**(2) Low polluting**
*Inv*	0.0732** (2.13)	0.0569 (1.32)
*Age*	−0.0050 (−0.95)	−0.0054*** (−4.62)
*Size*	0.6822** (2.23)	0.7795*** (3.28)
*Roe*	0.0142 (0.02)	−0.1459 (−0.59)
*Lev*	−0.0188** (−2.41)	−0.0071 (−0.80)
*Growth*	−0.1650** (−2.01)	0.0979 (0.67)
*First*	0.8667 (0.56)	−4.2974*** (−2.68)
*Cash*	−1.9095 (−1.54)	0.4997 (0.39)
*Year FE*	Yes	Yes
*Firm FE*	Yes	Yes
*R-squared*	0.3933	0.1871
*N*	569	553

(1) ** and *** represent significant at the 5 and 1% significance level, respectively.

*(2) *t*-values are provided in parentheses.*

The results show that cross-shareholding has a positive effect on corporate environmental investment in high polluting industry; however, the impact of cross-shareholding on corporate environmental investment is not observed in low polluting industry. Therefore, the positive impact of cross-shareholding on corporate environmental investment is more pronounced in the heavily polluting industry than in the low polluting industry, supporting *Hypothesis 3*.

### Robustness Checks

#### Alternative Measure of Explanatory Variable

To test the robustness of the baseline results, we use the crossholding scaled by total assets as the alternative measure of cross-shareholding, and re-estimate the main baseline regressions. The results in Table 5 show that the impact of cross-shareholding on environmental investment remains significantly positive when only controlling for annual dummy variable without additional control variable. As such, the results are consistent with the baseline results and the findings remain robust.

**TABLE 5:** Robustness checks for alternative measure.

**Variable**	**(1)**	**(2)**
*Inv*	0.0810*** (2.57)	0.0879*** (2.77)
*Age*		−0.0053*** (−4.39)
*Size*		0.8228*** (4.46)
*Roe*		−0.1021 (−0.44)
*Lev*		−0.0130** (−2.28)
*Growth*		−0.0675 (−1.04)
*First*		−1.7676* (−1.66)
*Cash*		−1.0021 (−1.14)
*Year FE*	Yes	Yes
*Firm FE*	Yes	Yes
*R-squared*	0.0160	0.2574
*N*	1121	1121

(1) *, **, *** represent significant at the 10, 5, and 1% significance level, respectively.

*(2) *t*-values are provided in parentheses.*

#### Random Sampling

To further test the robustness of the baseline results, we randomly select 1/2 of the total sample and re-estimate the main regressions. As shown in Table 6, the results are again consistent with the baseline results. Therefore, our baseline findings are robust and reliable.

**TABLE 6:** Robustness checks for random sampling.

**Variable**	**(1)**	**(2)**
*Inv*	0.1050*** (2.37)	0.0943** (2.19)
*Age*		0.0276*** (4.37)
*Size*		0.8600*** (3.47)
*Roe*		−1.4228** (−2.02)
*Lev*		−0.0567** (−2.50)
*Growth*		−0.0556 (−0.80)
*First*		−0.0362 (−1.01)
*Cash*		−0.00344 (−0.86)
*Year FE*	Yes	Yes
*Firm FE*	Yes	Yes
*R-squared*	0.0529	0.1842
*N*	560	560

(1) ** and *** represent significant at the 5 and 1% significance level, respectively.

*(2) *t*-values are provided in parentheses.*

## Conclusion

This article uses Chinese A-share listed firms from 2014 to 2019 as the research setting to investigate the impact of cross-shareholding on corporate environmental investment, and the results are summarized as follows. First, corporate participation in cross-shareholding will have a positive impact on firms’ environmental investment. Second, the positive impact of cross-shareholding on environment investment is more pronounced in state-owned firms or firms in high polluting industry. Third, the empirical results remain robust after using alternative measure of cross-shareholding and robust to random sampling.

Our findings have important implications for companies and policymakers. First, this article verifies that cross-shareholding contributes to corporate sustainable development by promoting environmental investment, providing insights for corporate sustainability. Second, State-owned firms and firms in high polluting industry can moderately increase their cross-shareholding to promote environmental investment. Third, although cross-shareholding benefits firms in terms of source allocation, strategic alliance, and profitability, the government should still be vigilant about this conduct, as the abuse of cross-shareholding between upstream and downstream firms can lead to industry monopoly, resulting in market disruptions that are detrimental to public welfare. Therefore, the government should strengthen the supervision and regulation to avoid malicious cross-shareholding practices.

## Data Availability Statement

The original contributions presented in the study are included in the article/supplementary material, further inquiries can be directed to the corresponding author/s.

## Author Contributions

JT: conceptualization, funding acquisition, project administration, and supervision. WC: investigation, validation, and writing–review and editing. XJ: formal analysis, methodology, and writing–original draft. All authors contributed to the article and approved the submitted version.

## Conflict of Interest

The authors declare that the research was conducted in the absence of any commercial or financial relationships that could be construed as a potential conflict of interest.

## Publisher’s Note

All claims expressed in this article are solely those of the authors and do not necessarily represent those of their affiliated organizations, or those of the publisher, the editors and the reviewers. Any product that may be evaluated in this article, or claim that may be made by its manufacturer, is not guaranteed or endorsed by the publisher.

## Footnotes

1
http://www.gov.cn/xinwen/2017-01/19/content_5161226.htm

2
https://www.mee.gov.cn/gkml/zj/bgt/200910/t20091022_174035.htm

3
https://www.mee.gov.cn/gkml/hbb/bgt/201712/t20171201_427287.htm

4
https://www.wind.com.cn/

5
http://cndata1.csmar.com/
